# Zika Virus Infection in the Central Nervous System and Female Genital Tract

**DOI:** 10.3201/eid2212.161280

**Published:** 2016-12

**Authors:** Emanuele Nicastri, Concetta Castilletti, Pietro Balestra, Simonetta Galgani, Giuseppe Ippolito

**Affiliations:** National Institute for Infectious Diseases Lazzaro Spallanzani, Rome, Italy (E. Nicastri, C. Castilletti, P. Balestra, G. Ippolito);; Azienda Ospedaliera San Camillo-Forlanini, Rome (S. Galgani)

**Keywords:** Zika virus, encephalopathy, female genital tract infection, viruses, central nervous system, Dominican Republic, Italy

**To the Editor:** On April 9, 2016, a 32-year-old woman from Italy traveled to Santo Domingo in the Dominican Republic. She worked as a volunteer nurse in the outpatient clinic of a primary school of a nongovernmental organization based in Italy. She returned to Italy on April 17. She did not have sexual intercourse during her stay abroad. 

On April 26, she was referred to the travel clinic of the National Institute for Infectious Diseases Lazzaro Spallanzani in Rome for a febrile syndrome with rash, generalized headache, and weakness, which started on April 21. Approximately 24 hours later, she was admitted to the institute’s medical facility for a suspected neurologic involvement. At admission, she had abnormal gait, strong asthenia, and a disseminated pruritic rash on her face, abdomen, chest, and arms, but she did not have a fever.

During physical examination, the patient was alert and fully oriented. Temperature was 36.9°C, pulse rate 90 beats/min, blood pressure 100/60 mm Hg, and respiratory rate 20 breaths/min. She had a diffuse erythematous macular rash and bilateral nonpurulent conjunctival hyperemia without meningeal signs. Findings of a neurologic examination of the upper limbs were within reference ranges.. Muscular strength was reduced in both legs (left > right), whereas tendon reflexes and all sensory modalities were within reference ranges. Results of a contrast-enhanced magnetic resonance imaging of the brain and spinal cord (on day 7), nerve conduction studies and electromyography (on day 8), and an electroencephalogram (on day 16) were within reference ranges. A lumbar puncture (on day 7) showed normal cell counts (<10 cells/mL), a normal glycorrachia/glycemia ratio (>0.5), and a slight increase in protein concentration (0.48 g/L [reference range 0.32−0.80 g/L]) in cerebrospinal fluid. Complete neuropsychologic examinations (on days 9 and 10) showed mild deficits in attention and mental processing speed and mental flexibility and moderate deficits in verbal and nonverbal memory tasks (Technical Appendix).

Real-time reverse transcription PCR (rRT-PCR) results for dengue viruses 1–4 and chikungunya virus were negative in serum and cerebrospinal fluid (CSF), whereas Zika virus RNA was detected in serum (day 7), urine (up to day 27), CSF (day 6), saliva (up to day 13), and vaginal swab (up to day 13) (Technical Appendix). Specific dengue and chikungunya IgG and IgM were not detected in serum and CSF. Zika virus IgM was detected in serum starting on day 6. Zika virus–specific antibodies in serum were confirmed by microneutralization assay (Table).

**Table:** Virologic test results during Zika virus infection in a 32-year-old woman after she returned from the Dominican Republic to Italy, April–June 2016*

Test/specimen type	1st sample, day 6†	2nd sample, day 7†	3rd sample, day 10†	4th sample, day 13†	5th sample, day 17†	6th sample, day 28†
Zika virus						
rRT-PCR‡ serum	Positive (32.9)	Negative	Negative	Negative	Negative	Negative
rRT-PCR‡ urine	Positive (34.2)	Positive (31.8)	Positive (32.4)	Positive (29.8)	Positive (32.1)	Positive (32.2)
rRT-PCR‡ saliva	ND	Positive (29.9)	Positive (33.5)	Positive (34.1)	Negative	Negative
rRT-PCR‡ CSF	ND	Positive (37.0)	ND	ND	Negative	ND
rRT-PCR‡ cervical swab sample	ND	Positive (31.1)	Negative	Positive (34.3)	Negative	Negative
IFA§ IgM titer	<1:20	1:40	1:160	1:80	1:320	1:1,280
IFA§ IgG titer	<1:20	<1:20	1:40	1:320	1:320	1:320
MNT¶ Ab titer	ND	ND	1:40	ND	1:160	>1:640
Dengue virus IFA§ IgM titer	<1:20	<1:20	<1:20	<1:20	<1:20	<1:20
Dengue virus IFA§ IgG titer	<1:20	<1:20	<1:20	<1:20	<1:20	<1:20
Chikungunya virus IFA§ IgM titer	<1:20	<1:20	<1:20	<1:20	<1:20	<1:20
Chikungunya virus IFA§ IgG titer	<1:20	<1:20	<1:20	<1:20	<1:20	<1:20

*Ab, antibody; CSF, cerebrospinal fluid; IFA, immunofluorescence assay; MNT, microneutralization test; ND, not done; rRT-PCR, real-time reverse transcription PCR.  †Days from symptom onset. ‡Zika virus–specific rRT-PCR (RealStar Zika Virus RT-PCR Kit 1.0; Altona Diagnostics GmbH; Hamburg, Germany). Numbers in parentheses indicate cycle threshold values (Technical Appendix). §IgG and IgM IFA (Arbovirus Mosaic 2; Euroimmun AG; Luebeck, Germany). Reference values (titer) serum: <1:20 = negative; >1:20 = positive (Technical Appendix). ¶MNT titers <1:20 were considered negative (Technical Appendix).

Starting on day 7, intravenous polyvalent immunoglobulins were administered (0.4 g/kg/day for 5 days); no adverse events were observed. A second neuropsychologic examination was performed on day 16 and indicated persistent impairment in memory performances and an improvement in mental concentration and flexibility tasks (Technical Appendix).

A second lumbar puncture (on day 17) showed an increased cell count (70 cells/mL, mostly lymphocytes), and CSF was negative for Zika virus RNA by rRT-PCR. The patient was discharged on day 20; she showed a progressive neurologic recovery starting on day 16. At 60-days follow-up visit, no neurologic deficits were reported.

During the 2013–2014 outbreak of Zika virus in French Polynesia and in the context of the 2015–2016 Zika virus circulation (*1*), an apparent increase in Guillain-Barré syndrome incidence was reported. Few anecdotal cases of encephalopathy in patients with Zika virus infection have been recently described in affected countries: 1 case in a man on a 4-week cruise through an area in the South Pacific that included New Caledonia, Vanuatu, the Solomon Islands, and New Zealand in 2015 (*2*); and 2 cases in Martinique (*3*) in February 2016. Recently, Zika virus has been detected in the genital tract of a virus-infected woman after Zika virus had disappeared from blood and urine (*4*), and a suspected case of Zika virus by sexual transmission from a woman to a man has been reported in New York City (*5*).

In our patient, Zika virus RNA was found in different systems, including the central nervous system and the genital tract. Recently, a mouse model of Zika virus infection by vaginal exposure demonstrated that Zika virus replicated within the genital mucosa, persisted postinfection, and was detected in the fetal brain of the mice (*6*). In our case, the patient reported early neurologic symptoms and moderate memory impairment in neuropsychologic examinations, all features consistent with the diagnosis of Zika virus–related encephalitis, which represents a rare atypical presentation, particularly in areas to which Zika virus infection is not endemic. A recent article shows that Zika virus can infect adult murine neural stem cells, leading to cell death and reduced proliferation (*7*). It raises the possibility that Zika is not simply a transient infection in adult humans and that exposure in the adult brain could have an effect on long-term memory or the risk for depression (*7*).

Our case highlights the potential for Zika virus neurotropism and the need for early identification of Zika virus–related neurologic symptoms. Moreover, the presence of Zika virus in the genital tract supports the recommendation of safe sex practice for women returning home from areas with ongoing Zika virus transmission.

Technical Appendix Methods for laboratory diagnosis and neuropsychologic examination.
