# Supplementary material for: Zika Virus Infection in the Central Nervous System and Female Genital Tract
Source: Emerg Infect Dis. 2016 Dec;22(12):2228–30. doi: 10.3201/eid2212.161280 (PMC5189169; doi:10.3201/eid2212.161280)
Supplement: Technical Appendix — Methods for laboratory diagnosis and neuropsychologic examination. [file 16-1280-Techapp-s1.pdf]

Article DOI: http://dx.doi.org/10.3201/eid2212.161280

Zika Virus Infection in the Central Nervous System and Female Genital Tract

Technical Appendix

Methods for Laboratory Diagnosis and Neuropsychologic Examination

Real-time RT-PCR for Detection of Zika, Dengue, and Chikungunya Virus RNA

Viral DNA was extracted from biological samples by using QIAsymphony technology (QIAGEN, Hilden, Germany) according to the manufacturer's instructions. Real-time reverse transcription PCR (RT-PCR) for the detection of Zika virus RNA (nonstructural protein 5 gene) was performed as described previously (*1*).

The CDC DENV-1-4 Real-Time RT-PCR Assay for Detection and Serotype Identification of Dengue Virus (http://www.cdc.gov/dengue/clinicalLab/realTime.html) was used to detect dengue 1-4 viruses RNA. To detect chikungunya virus RNA, a real-time RT-PCR targeting the envelope 1 structural protein gene was performed according to Pastorino et al. (*2*).

Serologic Assays

Serum samples were tested by indirect immunofluorescence assay for IgM and IgG against Zika virus, dengue virus, and chikungunya virus using the Arbovirus Mosaic 2 IgM and IgG (Euroimmun AG, Lübeck, Germany), according to the manufacturer's instructions.

The presence of Zika virus–specific neutralizing antibodies was confirmed by a microneutralization test (MNT) as described (*3*). MNTs were performed on heat-inactivated (at 56°C for 30 min) serum samples by exposing serial twofold dilutions of them in tissue culture medium (1:10–1:2,560) to 100 50% tissue culture infectious doses of Zika virus (INMI1 isolate strain). After incubation at 37°C for 30 min in 5% CO_2, 100 mL of the serum/virus mixtures was plated on each well of a 96-well plate covered by Vero E6 cell monolayers, and reincubated up to the appearance of an easy detectable cytopathic effect in control cultures. The dilution of serum able to reduce the cytopathic effect in 50% of infected cell cultures was determined. MNT titers <1:20 were considered negative.

Neuropsychologic Examination

A complete neuropsychologic examination was performed twice: the first test on day 8 and 9, and the second on day 15 (Technical Appendix Figure). Tests were selected to be sensitive to a

comprehensive range of different cognitive domains: mental flexibility (Trail Making Test-B; Stroop color-word, Controlled Oral Word Association), concentration and speed of mental processing (Trail Making Test-A; Wechsler Adult Intelligence Scale-Revised [WAIS-R] Digit Span, forward and backward; WAIS-R Digit Symbol; Corsi's Block-Tapping Test, Stroop word and color), memory (Rey Auditory Verbal Learning Test; Rey Complex Figure-delayed), visuospatial and constructional abilities (Rey Complex Figure-copy), and fine motor functioning (Grooved Pegboard Test, dominant/non dominant hand). The Beck Anxiety Inventory and Beck Depression Inventory II were administered.

References

1. Faye O, Faye O, Diallo D, Diallo M, Weidmann M, Sall AA. Quantitative real-time PCR detection of Zika virus and evaluation with field-caught mosquitoes. Virol J. 2013;10:311. PubMed http://dx.doi.org/10.1186/1743-422X-10-311

2. Pastorino B, Bessaud M, Grandadam M, Murri S, Tolou HJ, Peyrefitte CN. Development of a TaqMan RT-PCR assay without RNA extraction step for the detection and quantification of African chikungunya viruses. J Virol Methods. 2005;124:65–71. PubMed http://dx.doi.org/10.1016/j.jviromet.2004.11.002

3. Papa A, Perperidou P, Tzouli A, Castilletti C. West Nile virus—neutralizing antibodies in humans in Greece. Vector Borne Zoonotic Dis. 2010;10:655–8. PubMed http://dx.doi.org/10.1089/vbz.2010.0042

Figure

	April 29, 2016	May 6, 2016
**• Attention and speed of mental processing**		
Trail Making Test A	❖	□
WAIS-R Digit Span	□	□
WAIS-R Digit Symbol	□	□
Corsi's block tapping test	□	□
Stroop colour denomination	□	□
Stroop word lecture	□	□
**• Mental Flexibility**		
Trail Making Test B	❖	□
Stroop (Colour-Word)	❖	□
Controlled Oral Word (FAS)	❖	❖
**• Memory**		
Rey Auditory Verbal Learning (total)	◆	◆
Rey Auditory Verbal Learning (after 15')	◆	◆
Rey-Osterrieth Complex Figure (delayed)	◆	◆
**• Fine Motor Functining**		
Lafayette Grooved Pegboard Test (dominant hand)	❖	□
Lafayette Grooved Pegboard Test (non-dominant hand)	□	□
Finger Tapping Test	❖	□
**• Visuospatial and Costructional Abilities**		
Rey-Osterrieth Complex Figure (copy)	□	□
**• Self-report anxiety and depression questionnaires**		
Zung Self-rating Anxiety Scale	❖	□
Zung Self-rating Depression Scale	□	□

□ normal ❖ mild deficit ◆ moderate deficit

Technical Appendix Figure. Neuropsychologic tests and cognitive domains at baseline and during follow-up. WAIS-R, Wechsler Adult Intelligence Scale-Revised.
